# ^137^Cs in the meat of wild boars: a comparison of the impacts of Chernobyl and Fukushima

**DOI:** 10.1007/s10967-015-4417-6

**Published:** 2015-09-05

**Authors:** Georg Steinhauser, Paul R. J. Saey

**Affiliations:** Environmental and Radiological Health Sciences, Colorado State University, 1618 Campus Delivery, Fort Collins, CO 80523 USA; Institute of Radioecology and Radiation Protection, Leibniz Universität Hannover, Herrenhäuser Straße 2, 30419 Hannover, Germany; Vienna University of Technology, Atominstitut, Stadionallee 2, 1020 Vienna, Austria

**Keywords:** Fukushima, Chernobyl, *Sus scrofa*, Foodstuff, Food safety, ^137^Cs, Ecological half-life

## Abstract

The impact of Chernobyl on the ^137^Cs activities found in wild boars in Europe, even in remote locations from the NPP, has been much greater than the impact of Fukushima on boars in Japan. Although there is great variability within the ^137^Cs concentrations throughout the wild boar populations, some boars in southern Germany in recent years exhibit higher activity concentrations (up to 10,000 Bq/kg and higher) than the highest ^137^Cs levels found in boars in the governmental food monitoring campaign (7900 Bq/kg) in Fukushima prefecture in Japan. The levels of radiocesium in boar appear to be more persistent than would be indicated by the constantly decreasing ^137^Cs inventory observed in the soil which points to a food source that is highly retentive to ^137^Cs contamination or to other radioecological anomalies that are not yet fully understood.

## Introduction

In the course of the Chernobyl nuclear accident (April 26, 1986) and the Fukushima nuclear accident (March 11, 2011), large amounts of radionuclides have been released and deposited in the environment [1, 2]. The majority of the released activity was due to volatile radionuclides such as ^131^I, ^132^Te, ^134^Cs, and ^137^Cs. Following the Chernobyl nuclear accident, the importance of both the short and long-term health effects of releases of short-lived ^131^I into the environment has been recognized [3]. Longer-lived ^134^Cs and ^137^Cs, together with other long-lived, more refractory fission products such as ^90^Sr or actinides such as plutonium remain in the environment for a very long time after a nuclear accident. However, their emissions from Fukushima [4–9] did not compare nearly to the releases from Chernobyl [2, 10, 11]. Radiocesium exhibits a potential health threat, especially upon intake with contaminated food [12–17].

Food has been identified as a major contributor to the total radiation exposure after the nuclear accidents at Chernobyl and Fukushima [18, 19]. In our previous analysis of food monitoring data [19], we have identified wild boars (*Sus scrofa*) as hyperaccumulators of radiocesium from Fukushima. The purpose of this study is to compare published data from Europe and Japan for a juxtaposition of the impacts of Chernobyl and Fukushima on contamination levels in wild boars as well as to discuss possible radioecological implications of these data.

## Materials and methods

Existing data from food (or environmental) monitoring programs were used for this study. For the assessment of the Fukushima impact, MHLW data [19, 20] were used. Data for the Ukraine were taken from Gulakov [21]. For Germany, data were taken from LFU [22] and Semizhon et al. [23]. Austrian Data were taken from Sontag et al. [24] and AGES [25]. Data from Croatia were obtained from Sprem et al. [26].

Most of the Japanese monitoring data provided individual concentrations for ^134^Cs (*T*_1/2_ = 2.1 years) and ^137^Cs (*T*_1/2_ = 30.1 years). However, for the sake of better comparison, shorter-lived ^134^Cs was neglected in this study, as any ^134^Cs from Chernobyl has decayed below the detection limit already years ago. In some cases, the Fukushima data provided sum activity radiocesium only. In this case, the ^137^Cs activity concentration (A_Cs-137_) was calculated from Eq. 1 assuming a constant ^134^Cs/^137^Cs activity ratio of 0.98 [15] at the time of the accident (2011-03-11).

1 $$A_{{\rm{Cs}} {\text{-}} 137} = \frac{\varSigma }{{1 + \frac{{0.98 \cdot {\text{e}}^{{ - \lambda_{{\rm{Cs}} {\text{-}} 134} t}} }}{{1 \cdot {\text{e}}^{{ - \lambda_{{\rm{Cs}}{\text{-}} 137} t}} }}}}$$

In Eq. 1, Σ is the sum activity concentration of ^134^Cs + ^137^Cs given in the data base, *λ*_Cs-134_ the decay constant of ^134^Cs, *λ*_Cs-137_ the decay constant of ^137^Cs and *t* is the time that has elapsed between the accident and sampling.

## Results and discussion

### Comparison of activity concentrations

**Fig. 1:**
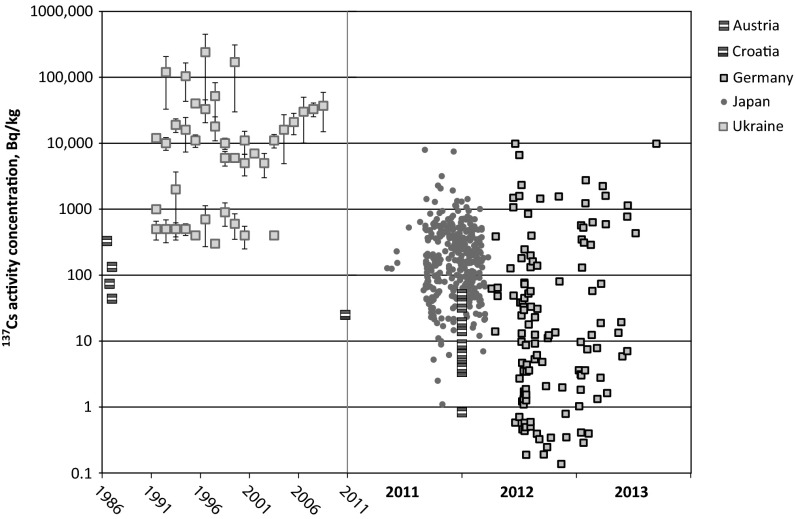
Activity concentrations in wild boar muscle tissue in Europe (Chernobyl) and Japan (Fukushima; data for the first year after the accident only), please note the different scales on the time axis. In case of averages instead of individual measurement data, the average is given with error bars corresponding to the respective uncertainties as given in the original publication. Activity concentrations are given in Bq/kg-wet at the time of sampling

A comparison of the ^137^Cs activity concentrations in wild boar muscle tissue is given in Fig. 1 (excluding the very recent results from Tanoi et al. [27]). After the Fukushima nuclear accident, the first exceedance of the 500 Bq/kg Japanese regulatory limit (sum of ^134^Cs + ^137^Cs) was reported in the course of the governmental food monitoring campaign in boar meat from Tochigi prefecture on July 16, 2011 (527 Bq/kg ^137^Cs). The maximum ^137^Cs activity concentrations, however, were observed in boar meat on September 5, 2011 and December 26, 2011 (7900 and 7500 Bq/kg, respectively; both from Fukushima prefecture) [19]. After April 1, 2012, the regulatory limit for regular food has been set to 100 Bq/kg in Japan. This limit has also been adopted by the European Union for imports from Japan. In many European countries including Germany and Austria, however, the regulatory limit for this type of food (and also from imports from Chernobyl-affected regions) has remained at 600 Bq/kg for radiocesium. It is important to note that the regulatory limit refers to the sum of ^134^Cs and ^137^Cs in both the European and Japanese legislation. Any ^134^Cs from Chernobyl (given its rather short physical half-life) has largely decayed away, whereas ^134^Cs from Fukushima is and still will be present for several years. It is also noteworthy that the ^134^Cs/^137^Cs activity ratio has been clearly distinct in both accidents: After the Chernobyl nuclear accident, ^134^Cs/^137^Cs activity ratios have been reported to be in the range of 0.5–0.6 [28–30], whereas ^134^Cs/^137^Cs activity ratio observed after the Fukushima nuclear accident was 0.98 [15] or “close to 1” [30].

This is the reason why a comparison of the compliance with the regulatory limits in Fig. 1 is difficult, because for Chernobyl-affected samples will only contain ^137^Cs, whereas Fukushima-affected samples will contain both ^134^Cs and ^137^Cs.

After the Chernobyl accident, boar meet from the vicinity of the NPP was contaminated to a much higher extent. Gulakov [21] reported on the maximum activity concentration amongst 3 wild boars from the “alienation zone” in 1996 of 661,000 Bq/kg, hence almost two orders of magnitude higher than levels found in Fukushima prefecture. It is remarkable, however, that this extraordinarily high value was found 10 years after the accident and not earlier. The levels observed in wild boars from the highly contaminated areas of Europe remained high for many years: even in the 2010s, wild boar meat from southern Germany (more than 1400 km away from the Chernobyl NPP site) occasionally exhibits ^137^Cs activity concentrations that are higher than those observed in Japan (see Fig. 1). Environmental agencies frequently report on boar meat in Central Europe the exhibits ^137^Cs activity concentrations that are in the range of 20 kBq/kg, which is a factor of 2–3 higher than the maximum values reported after Fukushima. It should also be noted that only selected boars among the wild boar population exhibit exorbitant activities, while others remain rather close to the detection limit. Hence there is a great variability which makes a complete assessment based on a limited number of samples difficult or impossible.

Data from independent researchers [27] studying wild boars from Japan and the distribution of radiocesium in their organs were largely in line with the results of the governmental food monitoring campaign. They found maximum radiocesium activity concentrations of 15,000 Bq/kg (approximately half of which is ^137^Cs). They also found the highest activity concentrations, as expected, in muscle tissue, but also in kidneys, tongue and heart tissue.

### Effective and ecological half-lives

Several studies reported on the time behavior of radiocesium contaminations in boar [21, 23]. It has become widely known that wild boars are not only hyperaccumulators of radiocesium, but also that the levels remain almost constant over long periods of time. Generally, the ecological half-life and/or the effective half-life are suitable measures to explain the behavior of radionuclides in various ecosystems and to predict future contamination levels. The effective half-life is defined by Eq. 2,

2 $$\frac{1}{{T_{\text{eff}} }} = \frac{1}{{T_{\text{eco}} }} + \frac{1}{{T_{\text{phys}} }}$$

where *T*_eff_ is the effective half-life,* T*_eco_ the ecological half-life and *T*_phys_ the physical half-life of ^137^Cs. According to Eq. 2, the effective half-life of a radionuclide in an ecosystem is always shorter than the physical half-life [31, 32], provided that no additional influx of radionuclides occurs to the ecosystem. In a previous publication [33], we have addressed the problem of the calculation of an effective half-life with increasing input of radioactivity (or before the “settling” of a release, allowing the processes that determine an ecological half-life to take place, such as migration, wash-out, uptake and removal by plants, fungi and other organisms etc.).

Based on the Fukushima data set we used for this study (comprising the first year after the accident), a calculation of the ecological half-life, therefore, is not feasible. It is unclear whether radiocesium activities have reached their maximum from where an effective half-life could be calculated. For relatively long-lived radionuclides, such as ^137^Cs a larger time-frame would be necessary to observe the decline in activity over several years.

However, the data for the Chernobyl accident allow for an estimation of the ecological half-lives of ^137^Cs observed in Europe. Based on data from southern Germany, Semizhon et al. [23] have concluded that the ^137^Cs levels found in wild boars have remained almost constant over the decade from 1998 to 2008. Indeed, we also observed constant activity concentrations for boar from southern Germany in the period of observation 2012–2015. In fact, the activity concentrations even exhibited a slight increase rather than a decline that would have been expected due to physical decay and environmental processes leading to a decline of activity.

**Fig. 2:**
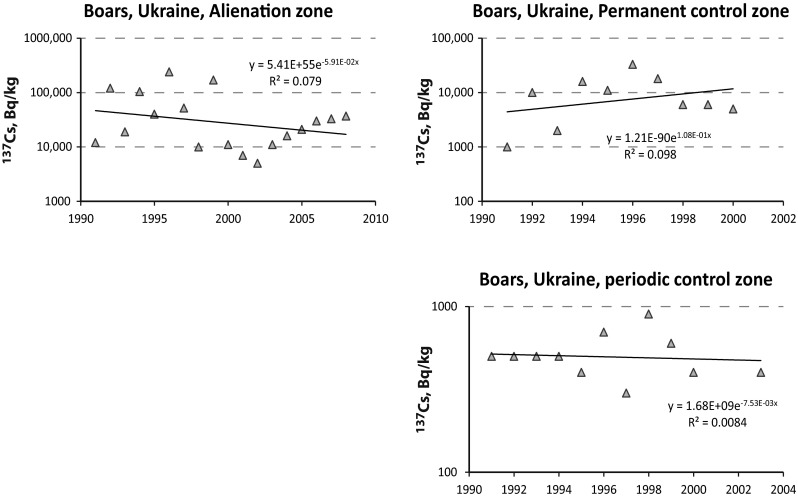
Analysis of data taken from Gulakov [21] for the estimation of the effective half-life of ^137^Cs in the meat of wild boars. Activity concentrations are given in Bq/kg-wet at the time of sampling

We observed a similar behavior in the data reported on contaminated areas around the Chernobyl exclusion zone [21] (Fig. 2): For the average data reported from boars in the “alienation zone”, we calculated an effective half-life of 11.7 years. In the somewhat less contaminated “permanent control zone”, no decline but again a slight increase in activities was observed (Fig. 2). In the “periodic control zone” a very slow decline is observed, corresponding to an effective half-life of 92 years, thus longer than the physical half-life of ^137^Cs. Pröhl et al. [34] reported an ecological half-life of ^137^Cs in boars as* T*_eco_ = 10.5 ± 1.6 years, which hence corresponds to an effective half-life of 7.8 years (Eq. 2).

**Fig. 3:**
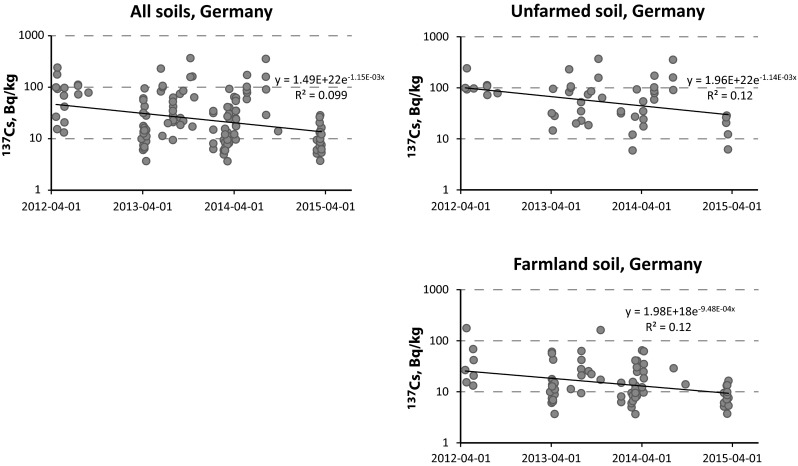
Effective half-lives of ^137^Cs observed in soil (all soils, farmland soil and unfarmed soil) in a 3-years period of observation in Bavaria (southern Germany). Activity concentrations are given in Bq/kg-dry at the time of sampling. Data taken from [22]

In any case, it is remarkable that the activity levels in wild boars remain rather constant, whereas these activity concentrations in soil decline comparably rapidly. Soil is the only primary reservoir of radiocesium for the wild boars’ fodder, hence this is a severe discrepancy. In southern Germany, we estimated the effective half-lives of ^137^Cs to be 1.7 years for unfarmed soil, 2.0 years for farmland soil, and an average 1.7 years for all soils in the database (see Fig. 3). Please note that the effective half-lives are based on 3 years of observation only and hence should be interpreted with care. Also it is unclear if the samples for this study by the LFU were taken in the same locations throughout this monitoring campaign. However, previous studies from the State of Upper Austria (close to the Bavarian border) also partly exhibited rather short effective half-lives, between 12 and 30 years. Interestingly, Austrian boar meat exhibits generally much lower activity concentrations than in neighboring Germany [25, 35].

Lastly, the question remains what the reason for this discrepancy is—long persistence in the “sink” (boar), though comparably short persistence in the primary “reservoir” (soil) in the environment. If the source gets constantly weaker, how can the sink retain its high levels over long periods of time? This is an obvious oxymoron to the expected decline of a radionuclide’s activity in both soil as well as boar due to decay and migration. One, it has to be acknowledged that soil is not the only reservoir, as there are intermediary organisms such as lichen or fungi which may act as soil-independent sources (secondary reservoirs) for the boars. Two, we hypothesize that the apparent time constancy of ^137^Cs in boar meat may, in part, be due to chemical changes in the deposited radiocesium. In particular, a partial transition of granular (thus insoluble) radiocesium to ionic and water soluble radiocesium may increase the bioavailability to fungi and fodder organisms of the boars and hence counterbalance the decline in absolute numbers of radiocesium atoms in the reservoir.

## Conclusions

 The impact of the Chernobyl nuclear accident on the ^137^Cs activities found in wild boars in Europe has been much higher than the impact of the Fukushima nuclear accident on wild boars in Japan. Although there is great variability within the ^137^Cs concentrations throughout the wild boar populations, some boars in southern Germany exhibit higher activity concentrations than the highest levels found in boars in Fukushima prefecture in Japan. Although we could prove that the radiocesium inventory in soil in southern Germany decreases constantly according to its effective half-live, the levels in boars appear to be much more persistent, if not constant. Currently, this oxymoron has not been fully resolved.
